# Endothelial specific YY1 deletion restricts tumor angiogenesis and tumor growth

**DOI:** 10.1038/s41598-020-77568-z

**Published:** 2020-11-24

**Authors:** Huan Liu, Yikai Qiu, Xiuying Pei, Ramamurthy Chitteti, Rebbeca Steiner, Shuya Zhang, Zheng Gen Jin

**Affiliations:** 1 Key Laboratory of Fertility Preservation and Maintenance of Ministry of Education, Department of Biochemistry and Molecular Biology, School of Basic Medical Sciences, Ningxia Medical University, Yinchuan, 750004 China; 2 Aab Cardiovascular Research Institute (CVRI), Department of Medicine, University of Rochester School of Medicine and Dentistry, 601 Elmwood Avenue, Box CVRI, Rochester, NY 14642 USA

**Keywords:** Tumour angiogenesis, Transcription

## Abstract

Angiogenesis is a physiological process for the formation of new blood vessels from the pre-existing vessels and it has a vital role in the survival and growth of neoplasms. During tumor angiogenesis, the activation of the gene transcriptions in vascular endothelial cells (ECs) plays an essential role in the promotion of EC proliferation, migration, and vascular network development. However, the molecular mechanisms underlying transcriptional regulation of EC and tumor angiogenesis remains to be fully elucidated. Here we report that the transcription factor Yin Yang 1 (YY1) in ECs is critically involved in tumor angiogenesis. First, we utilized a tamoxifen-inducible EC-specific YY1 deficient mouse model and showed that YY1 deletion in ECs inhibited the tumor growth and tumor angiogenesis. Using the in vivo matrigel plug assay, we then found that EC-specific YY1 ablation inhibited growth factor-induced angiogenesis. Furthermore, vascular endothelial growth factor (VEGF)-induced EC migration was diminished in YY1-depleted human umbilical vein endothelial cells (HUVECs). Finally, a rescue experiment revealed that YY1-regulated BMP6 expression in ECs was involved in EC migration. Collectively, our results demonstrate that endothelial YY1 has a crucial role in tumor angiogenesis and suggest that targeting endothelial YY1 could be a potential therapeutic strategy for cancer treatment.

## Introduction

Tumor angiogenesis is an initiated by series of events leading to tumor neovascularization, which maintains the tumor microenvironment during malignant tumor development and remodeling^1^. Various cell types in the tumor stroma including immune cells^2^, fibroblasts^3^, and endothelial cells (ECs)^4^ contribute to tumor angiogenesis and tumor growth. In response to pro-angiogenic stimuli, ECs play a key role in tumor angiogenesis. There are two distinct phenotypes of ECs, namely tip and stalk cells. EC tip cells lead vascular sprouting, extend filopodia and migration in response to vascular endothelial growth factor (VEGF), while EC stalk cells are highly proliferative and form the capillary lumen during angiogenesis in tumor tissues^5–7^. However, the underlying molecular mechanisms of tumor angiogenesis have not been fully elucidated.

Current literature suggests that the combinatorial regulation of EC transcription plays a crucial role in vascular network and maintenance of vascular integrity^8^. In particular, recently our group has revealed that Yin Yang 1 (YY1), a ubiquitously expressed GLI-Krüppel zinc finger-containing transcription factor^9,10^, regulates expression of angiogenic genes that are critical for proper vascular development and homeostasis^8^. Specifically, our group has reported that EC-specific YY1 deletion in mice led to embryonic lethality as a result of abnormal angiogenesis and vascular defects^8^. However, the role of endothelial YY1 in regulation of pathological angiogenesis has not been explored. It has been reported that YY1 in tumor cells was implicated in tumor angiogenesis through driving HIF1-dependent expression and secretion of VEGF in tumor cells^11–13^. Nevertheless, it remains unknown whether YY1 in ECs contributes to tumor angiogenesis and tumor growth.

To identify the role of endothelial YY1 in tumor angiogenesis and tumor growth, we generated tamoxifen-inducible EC-specific YY1-deficient (*YY1*^*iΔEC*^) mice for genetic ablation YY1 in ECs, and we found that EC-specific YY1 knockout in mice greatly diminished tumor angiogenesis and tumor growth in vivo.

## Results

### YY1 is highly expressed in human tumor endothelial cells

**Figure 1:**
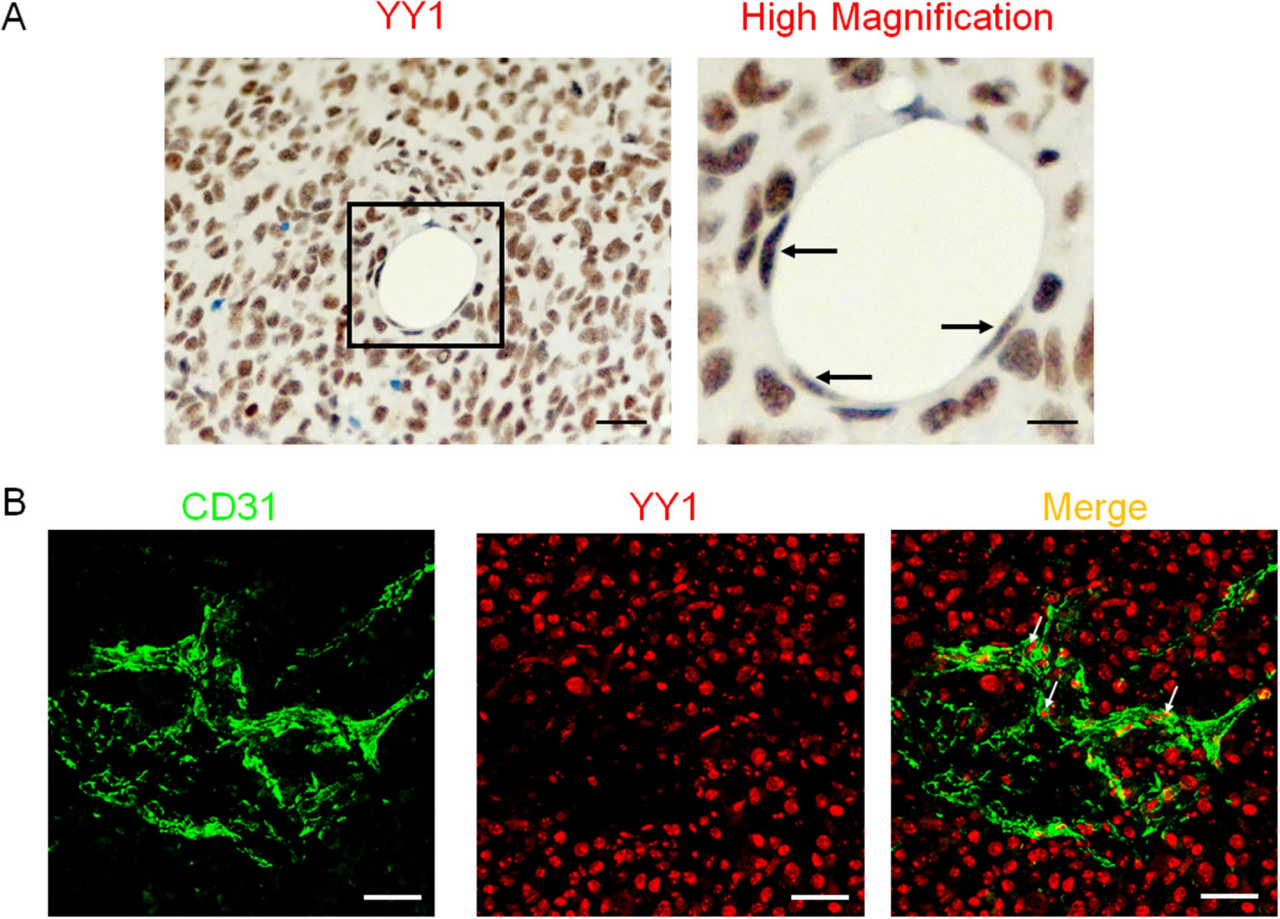
YY1 expression in tumor endothelial cells of human melanoma. (**A**) Human melanoma tissues were stained for YY1 antibody to localize protein expression. Scale bars = 200 µm. (**B**) Immunofluorescence confocal analysis of YY1 expression in tumor ECs of human melanoma tissues. scale bars = 50 µm.

To assess endothelial YY1 functional role in tumor angiogenesis, we initially determined the YY1 expression in tumor blood vessels of cancer tissue by immunohistochemistry. A significant level of YY1 expression was observed in both in tumor cells and tumor-associated ECs in human melanoma tissues (Fig. 1A). The expression of YY1 in melanoma ECs was further confirmed by immunofluorescence analysis using confocal microscopy (Fig. 1B). These results suggest that YY1 has potential role in regulation of tumor EC function.

### Characterization of endothelial specific YY1 knockout mice

**Figure 2:**
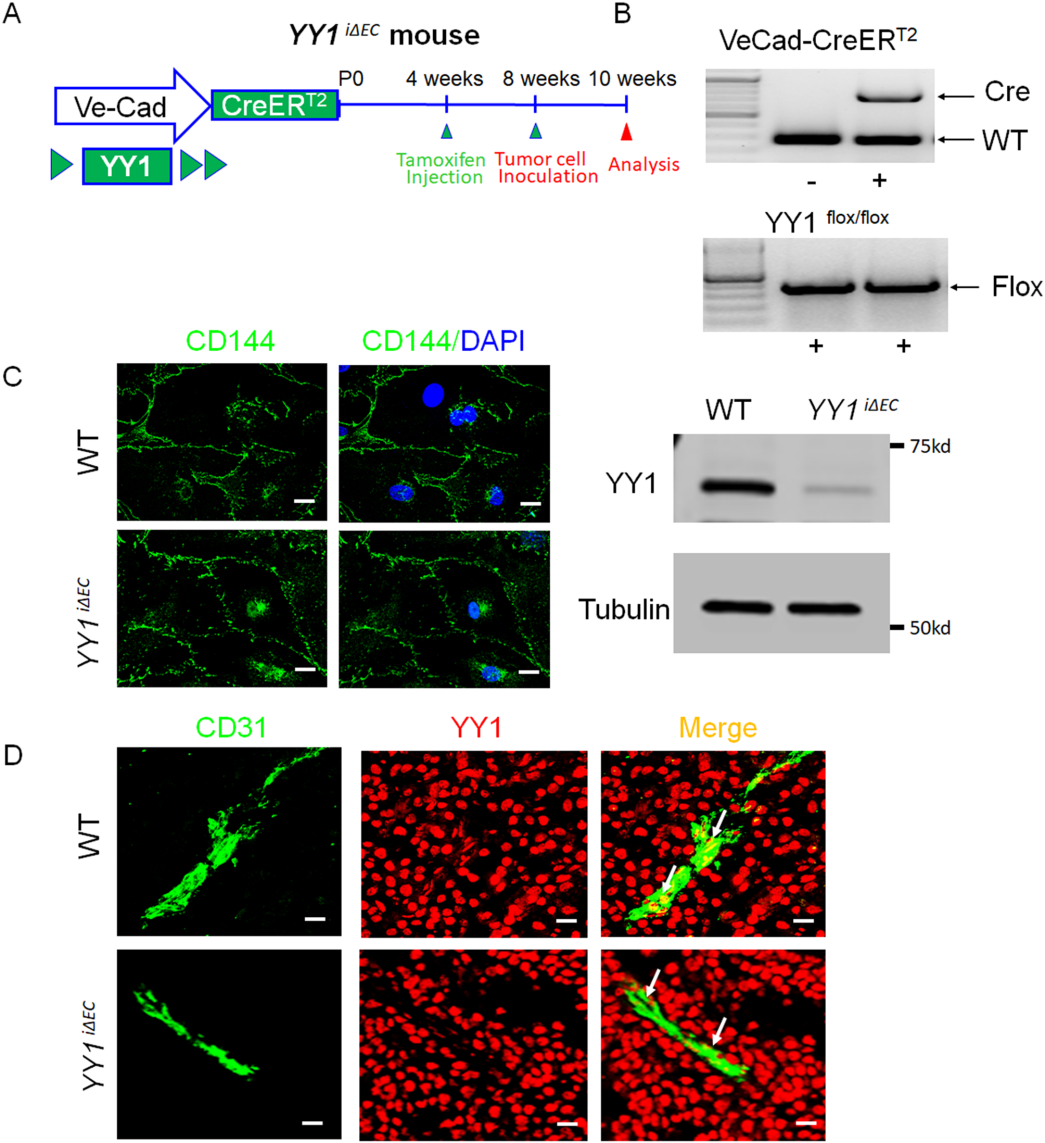
Generation and characterization of endothelial-specific YY1 deficient mice for the tumor angiogenesis. (**A**) Schematic diagram for the endothelial cell specific deletion of YY1 in mice (Ve-Cad-CreER^T2^; YY1^flox/flox^ , YY1^*iΔEC*^ ) and the strategy of the tumor angiogenesis study. The tumor was induced by melanoma B16-F10 cells (5 × 10^6^ cells per mouse) with subcutaneously transplanted into 8-week-old WT or *YY1*^*i∆EC*^ mice. (**B**) PCR analysis for the genotyping of Ve-Cad-CreER^T2^; YY1^flox/flox^ mice (YY1^*iΔEC*^) and YY1^flox/flox^ (WT) mice. (**C**) Immunofluorescence image of endothelial cell marker VE-Cadherin in lung endothelial cells isolated from YY1^*iΔEC*^ mice. Nuclei were labeled by DAPI (blue) (Left panel) and Western blot analysis of endothelial YY1 expression in mouse lung endothelial cells isolated from WT and *YY1*^*iΔEC*^ mice (n = 3) (Right panel). (**D**) Dual immunostaining analysis of YY1 (red) and endothelial cell marker CD31 (green) in melanoma tumor tissues isolated from WT and *YY1*^*iΔEC*^ mice, (n = 7). Scale bars: 20 μm.

To investigate the role of YY1 in tumor ECs in vivo, we generated EC-specific YY1 knockout mice by crossbreeding *YY1*^*flox/flox*^ with *Cdh5*-CreER^T2^ transgenic mice to create *YY1*^*iΔEC*^(*Cdh5*-CreER^T2^; *YY1*^*flox/flo*^) mice^14^. *Cdh5*-CreER^T2^ transgenic mice are well-established inducible gene knockout model for endothelium^15^. Expression of YY1 protein was explicitly depleted in ECs of *YY1*^*iΔEC*^ mice from the 4 weeks by injection of 66 mg/kg tamoxifen^8^ (Fig. 2A,B). YY1 gene knockout was also confirmed in isolated mouse lung ECs from *YY1*^*iΔEC*^ mice (Fig. 2C) and further confirmed with dual immunostaining of YY1 and EC specific CD31 marker in tumor tissue (Fig. 2D). These results clearly demonstrate EC-specific deletion of YY1 in *YY1*^*iΔEC*^ mice.

### *Specific deletion of YY1 in endothelial cells blocks tumor growth *in vivo

**Figure 3:**
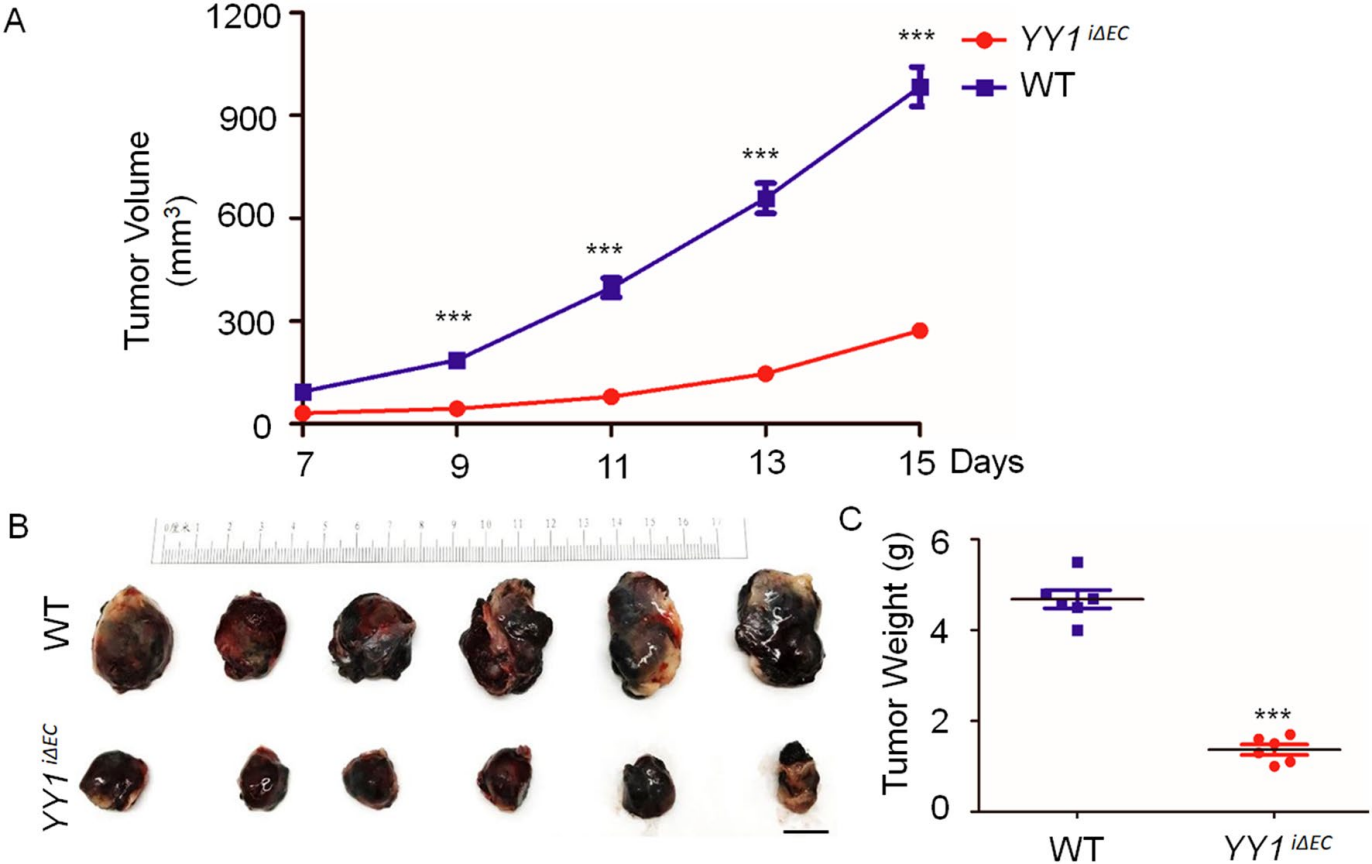
Endothelial-specific deletion of YY1 in mice reduces growth of implanted melanoma tumors. (**A**) Tumor growth curve by tumor volume shows a decrease in tumor volume in *YY1*^i∆EC^ mice compared to that in WT mice. (**B**, **C**) Tumor tissues were collected and analyzed after B16-F10 transplantation for the period of 15 days, Panel (**B**) shows solid tumor images and (**C**) shows the statistical data of tumor weight (n = 7). ** *p* < 0.01 vs. WT.

To investigate the effects of EC-specific deletion of YY1 on tumor growth, we inoculated melanoma B16-F10 cells on the dorsal side of 8-week-old mice in both YY1^i*∆EC*^ and WT mice (Fig. 2A). Tumor volume was quantified every 2 days for 15 days. Interestingly, tumor growth was drastically reduced in *YY1*^*i∆EC*^ mice (Fig. 3A) (< 50% of tumor volume compared to that in control littermates). The morphological analysis of tumor isolated from mice 15 days after B16-F10 cell transplantation showed that *YY1*^*i∆EC*^ mice had smaller tumor size (Fig. 3B) and significant reduction of tumor weight (Fig. 3C). Taken together, our results showed that EC-specific YY1 deletion in mice significantly suppressed tumor growth.

### Loss of YY1 in endothelial cells impairs tumor angiogenesis in mice

**Figure 4:**
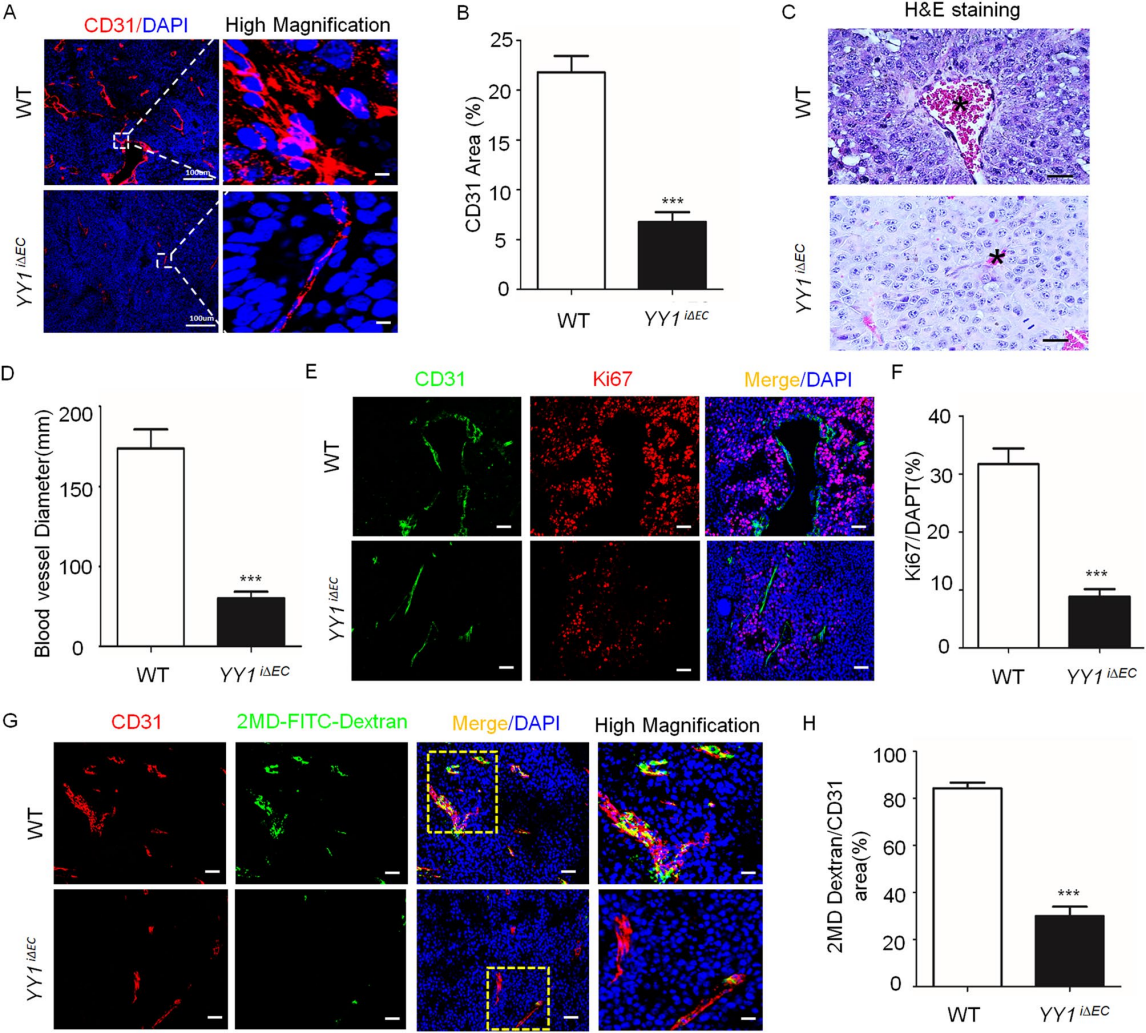
Endothelial *YY1* deletion in mice diminishes tumor angiogenesis. (**A**) Immunofluorescence staining of tumor tissue sections from WT or *YY1*^*i∆EC*^ mice with the antibody against endothelial cell marker CD31 (red). Nuclei were labeled by DAPI (blue). Scale bars: 100 μm. (**B**) Quantification of CD31 positive cells (five fields/group). (**C**) H&E staining for tumor tissue section from WT and *YY1*^*i∆EC*^ mice. Tumor vascular lumen (marked by *) in *YY1*^*i∆EC*^ mice was much smaller compared with those in WT mice, Scale bars:100 μm. (**D**) Quantification of blood vessel diameter (five fields/group). (**E**) Images of double immunofluorescence staining for blood vessels (CD31, green) and proliferating cells (Ki67, red). Nuclei were labeled by DAPI (blue). Scale bars:100 μm. (**F**) Quantification of Ki67-positive cells (five fields/group). (**G**) Analysis of tumor vessel blood perfusion by 2MDa FITC-dextran (green) via a tail-intravenous injection of tumor-bearing mice. Tumor tissue sections were co-stained with CD31 (red) and DAPI (blue). Scale bars:100 μm. (**H**) Histogram shows the average ratio of FITC-dextran/CD31 (five fields/group). Values represent mean ± SEM. Data was analyzed using unpaired Student’s t-test. **p < 0.01vs. WT.

To elucidate the cellular basis for the reduction of tumor growth and volume by endothelial-specific YY1 deletion, we focused on the neovascularization in tumor tissues. Using CD31 immunostaining, we observed that tumor angiogenesis in *YY1*^*i∆EC*^ was profoundly inhibited by the marked reduction of vascular capillary density (Fig. 4A,B). Tumor vasculature in WT mice consisted of vastly branched and tortuous blood vessels with a clear distinction of vascular sprouts. Whereas, tumor vasculature in *YY1*^*i∆EC*^ mice was less branched, less tortuous and reduced in diameter (Fig. 4B–D). Defective tumor capillaries manifested by CD31-postive staining and reduced cell proliferation evidenced by Ki67-postive staining were observed in tumor tissues from *YY1*^*i∆EC*^ tumors compared with those from WT mice (Fig. 4E,F). The quantification of vessel perfusion (marked by intravascular 2MD-FITC-Dextran) revealed a decrease of the functional vascular area in the tumors from *YY1*^*i∆EC*^ mice (Fig. 4G,H). These data indicate that endothelial YY1 critically regulates tumor angiogenesis.

### *Deletion of endothelial YY1 disrupts growth factors-induced angiogenesis in the matrigel plug model *in vivo

**Figure 5:**
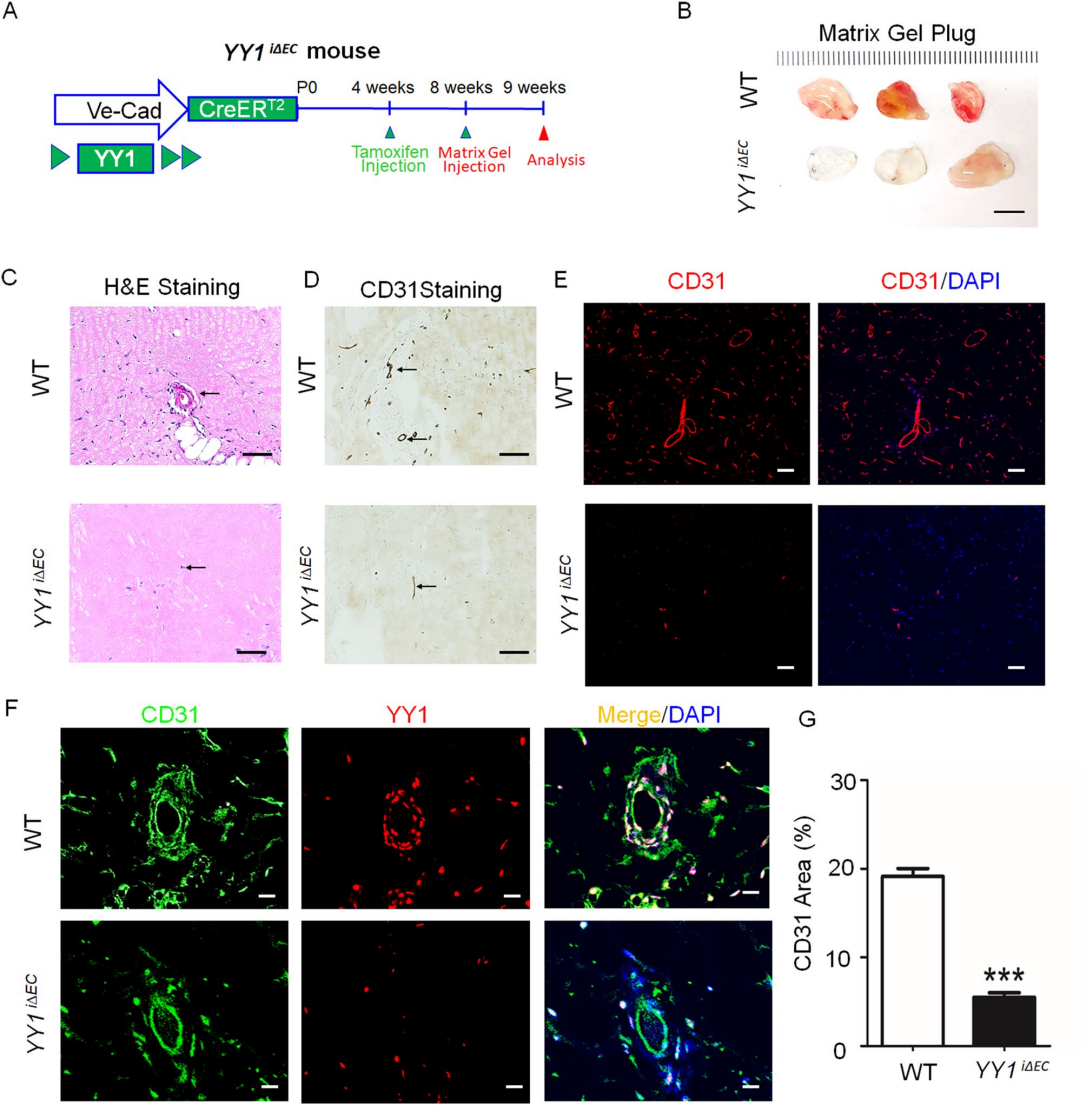
Matrigel plug assays substantiates that endothelial *YY1* is required for angiogenesis in vivo. (**A**) Schematic diagram for the EC-specific deletion of YY1 in mice from 4 weeks, matrigel injection at 8 weeks and angiogenesis analysis at 9 weeks using Ve-Cad-CreER^T2^; YY1^flox/flox^ (*YY1*^*i∆EC*^) mice and WT mice. (**B**) Image shows the morphology of the matrigel plugs harvested from *YY1*^*i∆EC*^ mice and WT mice. (n = 7). scale bar, 1.0 cm. (**C**) H&E staining for matrigel plug sections from WT and *YY1*^*i∆EC*^ mice. Vascular lumen (marked by ↑) in the matrigel plugs from *YY1*^*i∆EC*^ mice was much smaller compared with those from WT mice, Scale bars:100 μm. (**D**, **E**) Histochemical and immunofluorescent staining for matrigel plug section from WT and *YY1*^*i∆EC*^ mice. CD31^+^ cells (marked by ↑) in the matrigel plugs from *YY1*^*i∆EC*^ mice was much fewer compared with those from WT mice, Scale bars:100 μm. (**F**) Dual immunostaining analysis of YY1 (red) and endothelial cell marker CD31 (green) in the sections of the matrigel plugs from WT and *YY1*^*iΔEC*^ mice, Nuclei labeled by DAPI (blue) (n = 7). Scale bars: 20 μm. (**G**) Quantification of CD31-positive cells (five fields/group). Values represent mean ± SEM. Data was analyzed using unpaired Student’s t-test. ** *p* < 0.01vs. WT.

To substantiate the functional role of endothelial YY1 in angiogenesis, we performed the in vivo matrigel plug assay. This model is a well-established model for identifying blood vessel formation as well as the functional assessment of endothelial cell migration in vivo. The matrigel comprising of VEGF and fibroblast growth factor (bFGF) was inserted on the dorsal side of 8-week-old *YY1*^*i∆EC*^ mice and WT mice. This experimental condition was maintained for a period of 7 days (Fig. 5A).VEGF-induced angiogenesis on the matrigel plugs of *YY1*^*i∆EC*^ mice had a paler appearance and less microvessels than WT control (Fig. 5B). Histological observations by H&E staining showed fewer blood vessels in the matrigel plugs of *YY1*^*i∆EC*^ mice (Fig. 5C). Capillary density in marigel plugs was visualized by using CD31 immunostaining. The number of CD31 positive vascular structures were significantly lower in *YY1*^*i∆EC*^ group than WT control group (Fig. 5D,E). The results were consistently correlated with dual immunostaining of CD31 and YY1 in the microvessels of matrigel plugs from *YY1*^*i∆EC*^ mice and WT controls (Fig. 5F,G). The results strongly indicate that EC-specific loss of YY1 attenuates growth factors-mediated EC migration and vascular invasion on the matrigel plugs and suppress growth factors-mediated angiogenesis in vivo.

### *Loss of endothelial YY1 alters endothelial gene expression and limits VEGF-induced EC migration *in vitro

**Figure 6:**
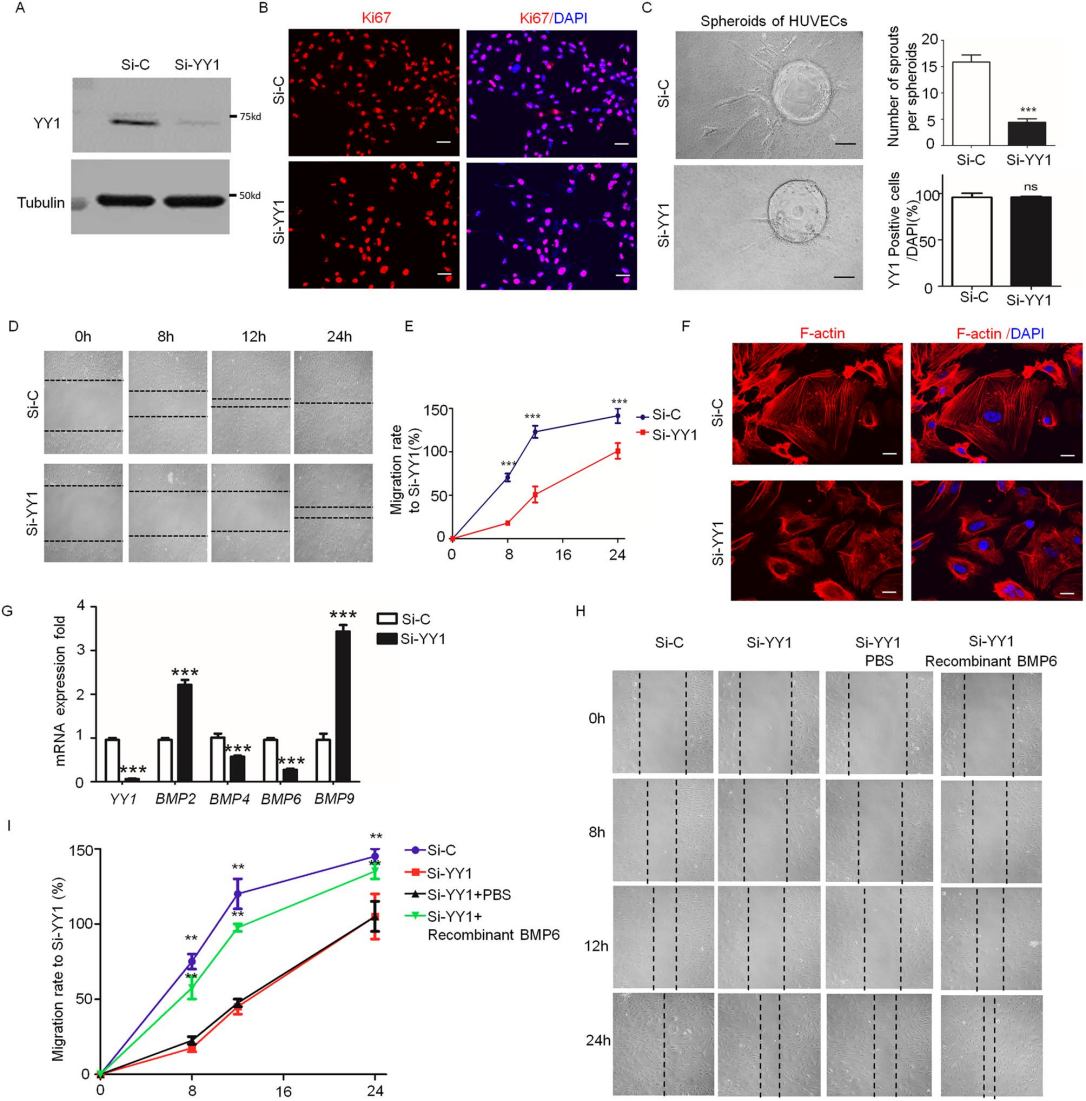
YY1 depletion by siRNA in endothelial cells alters gene expression and impairs cell migration. (**A**) Western blot confirms reduced expression of YY1 in HUVECs treated with YY1 siRNA (Si-YY1) and control siRNA (Si–C) for 24 h. (**B**) Immunofluorescence staining of HUVEC treated with YY1 siRNA and control siRNA for 24 h and then staining with proliferation marker Ki67 (red). Nuclei labeled by DAPI (blue). Scale bars: 100 μm. (**C**) The morphology of HUVEC treated with YY1 siRNA or control siRNA for 24 h and then conjugated to spheroids under the stimulus of the conditional medium from B16 cells after 3 days, Scale bars: 100 μm. (**D**) Endothelial migration of VEGF-induced HUVECs treated with or without Si-YY1 after 8 h, 12 h and 24 h, n = 3, VEGF (20 ng/ml). (**E**) Statistical results of HUVEC migration rates. **P < 0.01 Si-YY1vs. Si–C.HUVECs treated with control siRNA andYY1 siRNA for 24 h. (**F**) Immunofluorescence staining of HUVEC treated with YY1 siRNA or control siRNA for 24 h under VEGF stimulation and then staining with F-actin (red). Nuclei labeled by DAPI (blue). Scale bars: 20 μm. (**G**) Quantitative real-time PCR analysis shows that YY1 depletion alters BMP family gene expression in the HUVECs, which treat with Si–C or Si-YY1 after 24 h. (**H**) The endothelial migration of HUVEC treated with YY1 siRNA or control siRNA for 24 h and then treatment with VEGF (20 ng/ml) and added PBS or Recombinant Human BMP6(20 ng/ml), the data collect at 8 h, 12 h and 24 h. (n = 3). (**I**) Statistical results of HUVEC migration rates. **P < 0.01 Si-YY1vs. Si–C.HUVECs treated with control siRNA andYY1 siRNA for 24 h.

To elucidate the molecular mechanisms by which YY1 regulates tumor angiogenesis, we used siRNA-mediated YY1 knockdown in human endothelial cells (HUVECs). YY1 knockdown in HUVECs after siRNA treatment was confirmed by Western blot (Fig. 6A). The loss of YY1 did not affect EC proliferation as indicated by Ki67 staining (Fig. 6B). However, we noticed an obvious decrease in the sprouting ability of YY1-depleted HUVEC spheroids with the supplementation of the conditional medium from cultured B16-F10 cells (Fig. 6C). In order to assess VEGF-mediated EC migration, we performed a wound healing assay. The cell migration results showed that the YY1 knockdown in HUVECs significantly blocked VEGF-induced endothelial migration (Fig. 6D,E). To determine the effect of YY1 depletion on cytoskeleton remodeling, F-actin staining was performed. Slight modifications were observed in F-actin levels in YY1 knockdown ECs (Fig. 6F). The comprehensive gene expression patterns of YY1 siRNA-treated HUVECs and control siRN-treated HUVECs were analyzed by Affymetrix Microarray^8^. The microarray data showed that YY1 depletion in ECs altered sets of genes involved in cell migration. Further validation of microarray results with qPCR analysis confirmed that there was a significant reduction of *BMP4* and *BMP6* expression and an increase of *BMP2* and *BMP9* expression in YY1-depleted ECs (Fig. 6G). To establish a correlation between the BMP pathway and YY1 during EC migration, we performed rescue experiments in the presence of BMP6 in YY1 siRNA-treated HUVECs. Recombinant BMP6 protein partially restored cell migration ability of YY1 siRNA-treated HUVECs (Fig. 6H,I). The results suggested that a decrease of BMP6 in YY1 siRNA-treated HUVECs could contribute to the impairment of endothelial migration.

## Discussion

In the present study, we demonstrate a specific role of endothelial YY1 in promoting tumor growth and tumor angiogenesis in vivo. Furthermore, we reveal that endothelial YY1 knockdown inhibits VEGF-induced EC migration and angiogenesis. Our findings indicate a critical role of endothelial-specific YY1 in tumorigenesis and suggest endothelial YY1 as a potential target for limiting tumor angiogenesis.

YY1, a ubiquitously expressed and multifunctional transcription factor, has been implicated in various aspects of tumor growth. In cancer cells, YY1 promotes cell cycle-related gene expression and promotes cell proliferation and invasion^11^. The silence of YY1 decreased cell growth in adherent, semisolid condition as well as adhesion to substrates, specifically collagen^16^. The functional role of YY1 in tumor angiogenesis is reported in various tumor tissues including the brain, hepatocellular carcinoma^17^, and B-cell lymphomas^11^. The reduction of YY1 in osteosarcoma cells can interfere with their metastatic implantation and angiogenesis^11^. YY1 in tumor cells enhances tumor angiogenesis by binding with the promotor of VEGFα and augmenting transcriptional activity in tumor cells^17^. These reports denote that the YY1 is necessary to tumor cell invasion, adhesion, metastasis, and migration. In this study, we have added substantial evidence supporting that endothelial YY1 has a critical role in promoting tumor angiogenesis and tumor growth. Specifically, by using the tamoxifen induced EC-specific YY1 knockout mice, we showed that endothelial YY1 deletion significantly suppress tumor angiogenesis and tumor growth.

Moreover, using the matrigel plug assay, we also showed that YY1 deletion in ECs blocked growth factors-induced EC migration and angiogenesis in vivo. In addition to this, using the culture cell system, we showed that the knockdown of endothelial YY1 by siRNA attenuated VEGF-induced ECs migration in vitro. The molecular pathways underlying these consequences of endothelial YY1 deficiency on EC angiogenetic function remain unclear. It has been reported that YY1 inhibits Notch signaling by binding to the ANK domain of Notch1 receptor^18,19^. YY1 silencing has been shown to interfere with the CXCR4/angiogenesis pathway^11^. We have recently uncovered that YY1 directly interacts with RBPJ in ECs to regulate endothelial sprouting and angiogenesis^8^. From our gene array results, we noticed that endothelial YY1 regulates many angiogenetic genes such as BMP family genes that are involved in regulation of EC migration and cell matrix remodeling. The rescue experiment confirmed that BMP6 is involved in YY1-mediated EC migration. Additional studies are required to further clarify the molecular pathways behind the specific effect of YY1 on gene expression in tumor ECs during tumor angiogenesis.

The specified multifunctional role of YY1 in tumor angiogenesis points to YY1 being a good candidate to be targeted for cancer therapy. The tumor microenvironment includes tumor cells, secreted proteins, and blood vessels^20^ are implicated in tumor formation and development. Earlier studies showed that the YY1 specific deficiency in tumor parenchyma cells suppress tumor angiogenesis in several types of tumors such as hepatocellular carcinoma (HCC)^17^ and prostate cancer^21^. The present study revealed that ECs as significant composition in the tumor microenvironment and substantiates the beneficial effects of YY1 depletion against tumor growth. Indeed, the physiological action of ECs is needed in the initial phase of tumor growth in order to sustain nutrient-rich microenvironment and tumor growth and hence plays a key part in tumor angiogenesis^22^. In this study, reduced tumor growth in *YY1*^*iEC*^ mice was associated with a diminishment of tumor angiogenesis. A probable cause could be attributed the reduced migration of ECs into tumor tissue as evidenced by the experiments with the in vivo Matrigel plug angiogenesis assay. The present investigation opens a window for therapeutic intervention with pharmacological targeting of YY1. Intriguingly, it has been reported that nitric oxide^23^ and rituximab^24^ inhibit YY1 expression in human tumor cells. However, it needs to be examined whether these drugs could be applied to specifically target YY1 in ECs and tumor angiogenesis.

In summary, this study uncovers the unique function of endothelial-specific YY1 in promoting tumor angiogenesis and tumor growth.

## Materials and methods

### Mice and treatments

All animal procedures were carried out in accordance with the Guideline for the Care and Use of Laboratory Animals published by the. National Institutes of Health, USA and were approved by the Institutional Animal Care and Use Committee, University of Rochester Medical Center. To evaluate the potential effect of YY1 deletion in ECs on the tumor model, endothelial cell-specific YY1 deficient (*YY1*^*flox/flox*^; VeCad-CreER^T2^, *YY1*^*iΔEC*^ ) mice were created by crossing *YY1*^*flox/flox*^ mice with VeCad-CreER^T2^ mice. The conditional knockout YY1 (*YY1*^*flox/flox*^) mice^25^ was acquired from Jackson Laboratory. VeCad-CreER^T2^mice^14^ was obtained from Ralf Adams, the University of Münster under the Material Transfer Agreement. *YY1*^*flox/flox*^ mice were manipulated as littermate wild type (WT). 4-week-old *YY1*^*iΔEC*^ mice were administration of tamoxifen using the following schedules and dosages: 66 mg/kg of tamoxifen was intraperitoneally injected over 5 consecutive days starting from 30 days of age^25^. The mice were genotyped by DNA extracted from the tail^14^. Primer sequences: YY1 flox: Forward ACCTGGTCTATCGAAAGGAGCAC; Reverse GCTTCGCCTATTCCTCGCTCATAA; VeCad-CreER^T2^: Forward CTGGGATGCTGAAGGCATCAC; Reverse TTGCGAACCTCATCACTCGTT.

### Tumor angiogenesis

8 weeks YY1^WT^ or *YY1*^*iΔEC*^ mice were anesthetized with ketamine/xylazine (100/20 mg/kg). Doral fur in t was removed by using a fur trimmer (Wahl clipper corporation, Sterling) and the skin cleaned with 75% ethanol. 1 × 10^6^ mouse melanoma cells (B16F10) in 100 µl PBS was injected subcutaneously of using a 1 ml syringe with a 25-gauge needle^17^. After 15 days, mice were sacrificed, and tumors tissue were collected for imaging, weight measurement, and histological analysis.

### *Matrigel plug *in vivo* angiogenesis assay*

YY1^WT^ or *YY1*^*iΔEC*^ mice at 8 weeks of age were anesthetized with ketamine/xylazine (100/20 mg/kg) and the skin was cleaned with 75% ethanol. Matrigel along with 500 ng/ml VEGF and 250 ng/ml fibroblast growth factor (bFGF) respectively was subcutaneously inserted in to each mouse. 7 days after the experiment mice were sacrificed, and tumor were collected for image, weight and histology analysis^26^.

### Histology and immunohistochemistry

Paraffin-embedded tumor sections were stained with hematoxylin and eosin (H&E) for cell morphologic evaluation (Darmstadt, Cat No.PS103-01). Sections were imaged with a BX51 light microscope and analyzed using spot 5.0 software. For analysis of tumor blood vessel were perfused with 2MDa FITC-dextran (green) via a tail-intravenous injection of tumor-bearing mice. For immunofluorescence studies in tumor tissues, fixed frozen sections were blocked with 10% goat serum albumin, and incubated overnight with the following primary antibodies: CD31(1:50, Abcam, ab28364); α-SMA (dilution 1:100; Cat No. M0851 Dako), Ki67 (1:100; Abcam, ab16667), Ve-cadherin (1:50; eBioscience, 14–1449-82) and YY1 (dilution 1:100; Cat No.Ab109231, Abcam). For unconjugated antibodies, appropriate secondary antibodies (dilution 1:500; Sigma-Aldrich, Cat No.ab1507, Taufkirchen) were added on the following day, before mounting with Prolong Gold anti-fade mounting media with DAPI (Thermo Fisher, Cat No.D1306).

### Isolation of mouse lung endothelial cells

Mouse lung ECs were isolated as previously described protocol with minor modifications^27^. Briefly, mouse lungs were dissected in ice-cold PBS and digested in a mixture of collagenase type I (3 mg/ml, Worthington), DNase I (Sigma-Aldrich) and dispase (Invitrogen) for 40 min at 37 °C. ECs were then separated using Dyna beads (Invitrogen) coated with anti-PECAM-1 antibody and cultured in DMEM medium (ATCC/30–2002) supplemented with 20% fetal bovine serum (FBS, Gibco) and EC growth supplement (BD Biosciences). Confluent ECs were trypsinized and separated using Dyna beads coated with anti-ICAM-2 antibody (BD Biosciences). Following two rounds of sorting, when the purity of ECs reached over 90%, the cells were used (within two passages of their initial isolation).

### Cell culture experiments

Primary cultured human umbilical vein endothelial cells (HUVECs) were purchased from Cell Applications, INC. The HUVECs were cultured in Medium 200 with 1X low-serum growth supplement (LSGS) in 10 mm dishes pre-coated with 0.1% gelatin as previously described^28^. HUVECs at passages 3–6 were used for this study.

### siRNA transfection

HUVECs that reached more than 80% confluence in 60-mm dishes were used for transfection. In brief, RNAiMax transfecting agent (6 µl; Invitrogen; Cat No.13–778-030) was mingled with Opti-MEM (250 µl; Invitrogen; Cat No.11–058-021), and then Non-targeting Control SMARTpool of control siRNAs (Catalog #:D-001810–10-05, GE Healthcare), or SMARTpool: ON-TARGET^plus^ YY1 siRNA (Catalog #: L-011796–00-0005, GE Healthcare) were diluted in 250 µl Opti-MEM, mixed gently, and incubated at room temperature for 20 min. A total of 0.5 ml of this mixture was added to HUVECs in 1.5 ml Opti-MEM and incubated for four hours. Then the media was replaced with completed Medium 200 and cells were treated after 48 h after transfection^10^.

### Wound healing assay

HUVECs were transfected with siRNA to knockdown YY1 gene and then the cells were stimulated by conditional medium from B16-f10 or VEGF (20 ng/mL). A micropipette tip was used to scrape a straight line in each well. After 8, 24 and 48 h, the migration of cells was analyzed by comparing the wound distance ratio starting from 0hrs to 24 h. Each experiment was performed three times^17^.

### Western blot analysis

The isolated mouse lung ECs and total cell lysates were harvested in freshly-prepared lysis buffer (20 mM Tris–HCl pH 7.5, 150 mM NaCl, 1% Triton X-100, 1 mM EDTA, 1 mM EGTA, 2.5 mM sodium pyrophosphate, 1 mM β-Glycerolphosphate, 50 mM NaF, 1 mM Na3VO4, and 1% protease inhibitor cocktail). After clarification at 4 °C, the cells were spun down at 12, 000 g for 15 min; total cell lysate was collected for SDS-PAGE gel analysis. After a 1.5 h transfer at 250 mV, the membranes were blocked in LI-COR blocking buffer diluted 1:1 with PBS at room temperature for one hour. Then the blots were incubated with primary antibodies YY1 (dilution 1:1000; Cat No.Ab109231, Abcam) and Tubulin (dilution 1:1000; Cat No.Ab6046, Abcam) diluted in 3% BSA at 4 °C overnight, followed by incubation with LI-COR IRDye 680RD goat anti-mouse IgG (H + L) or IRDye 800CW goat anti-rabbit IgG (H + L) or IRDye 680RD donkey anti-goat IgG (H + L) (dilution at 1:10,000) at room temperature for 30 min. Images were visualized using an Odyssey Infrared Imaging System (LI-COR)^29^. Densitometry analysis of blots was performed using NIH Image J software (ImageJ bundled with 64-bit Java 1.8.0_112, http://imagej.nih.gov/ij/).

### Quantitative real-time PCR

After treatment, total RNA was extracted using a QIAGEN RNeasy Mini kit (Qiagen, Cat No.74136)^29^. RNA concentration and purity were determined by Nanodrop2000 Spectrophotometer (Thermo Fischer Scientific). For reverse transcription, 0.5–1 µg of total RNA was converted first to strand complementary DNA (cDNA) using a High-Capacity cDNA Reverse Transcription Kit (Applied Biosystems, Cat No. 4374966) following the manufacturer's instructions. Quantitative real-time PCR was then performed with a Bio-Rad iQ5 real-time PCR thermal cycler, using iQ SYBR Green Supermix (Bio-Rad, Cat No. 1708886) for relative mRNA quantification. All primer sequences were listed in Table S. The comparative cycle threshold (Ct) method (2 − ΔΔCt) was used to determine the relative mRNA expression of target genes after normalization to the housekeeping gene GAPDH or β-actin.

### Statistical analysis

Values are presented as mean ± SD. Statistical analysis was performed using Graph Pad Prism (GraphPad Software, Version 7.0, https://www.graphpad.com/demos/). Results were evaluated by t-test or by one- or two-way analysis of variance (ANOVA) when appropriate. A *P* value *P* < 0.05 was statistically significant.

## Supplementary information

Supplementary Information 1.

## Abbreviations

bFGF: Basic fibroblast growth factor
ECs: Endothelial cells
HCC: Hepatocellular carcinoma
H&E: Hematoxylin and eosin
HUVECs: Human umbilical vein endothelial cells
*YY1*^*iΔEC*^: Tamoxifen-inducible EC-specific YY1-deficient
VEGF: Vascular endothelial growth factor
WT: Wild type
YY1: Yin Yang 1
