# Supplementary material for: Endothelial specific YY1 deletion restricts tumor angiogenesis and tumor growth
Source: Sci Rep. 2020 Nov 24;10:20493. doi: 10.1038/s41598-020-77568-z (PMC7686504; doi:10.1038/s41598-020-77568-z)
Supplement: Supplementary file 1 — Supplementary Information 1. [file 41598_2020_77568_MOESM1_ESM.docx]

**Supplementary information**

**Endothelial Specific YY1 Deletion Limits Tumor Angiogenesis and Tumor Growth**

Huan Liu ^1,2^, Yikai Qiu^1^, Xiuying Pei ^1^, Ramamurthy Chitteti^2^, Rebbeca Steiner^2^, Shuya Zhang ^1,2,*^，Zheng Gen Jin ^2,*^

^1^ Key Laboratory of Fertility Preservation and Maintenance of Ministry of Education, Department of Biochemistry and Molecular Biology, School of Basic Medical Sciences, Ningxia Medical University, Yinchuan, China.

^2^Aab Cardiovascular Research Institute, Department of Medicine, University of Rochester School of Medicine and Dentistry, Rochester, NY, USA

Table S Primers used in RT-PCR for expression analysis of the BMP family gene expression

| **Gene** | **Direction** | **Sequence (5’- 3’)** | **Length(nt)** | **Tm ( ^o^C)** |
| --- | --- | --- | --- | --- |
| *YY1* | Forward | GGATAACTCGGCCATGAGAAA | 21 | 62 |
|  | Reverse | GAAAGGGCTTCTCTCCAGTATG | 23 | 62 |
| *BMP2* | Forward | CAGCTGTAAGAGACACCCTTTG | 22 | 63 |
|  | Reverse | GCATTCTCCGTGGCAGTAAA | 20 | 63 |
| *BMP4* | Forward | GGGAGAAGCAGCCAAACTAT | 20 | 62 |
|  | Reverse | CTTGAGGTAACGATCGGCTAAT | 22 | 62 |
| *BMP6* | Forward | GTGCGCCAACTAAGCTAAATG | 21 | 62 |
|  | Reverse | TGGCATCCACAAGCTCTTAC | 20 | 62 |
| *BMP9* | Forward | CAACAGGTACACGTCCGATAAG | 22 | 62 |
|  | Reverse | GAAGTCCTCTGTGGCAGTTATG | 22 | 62 |


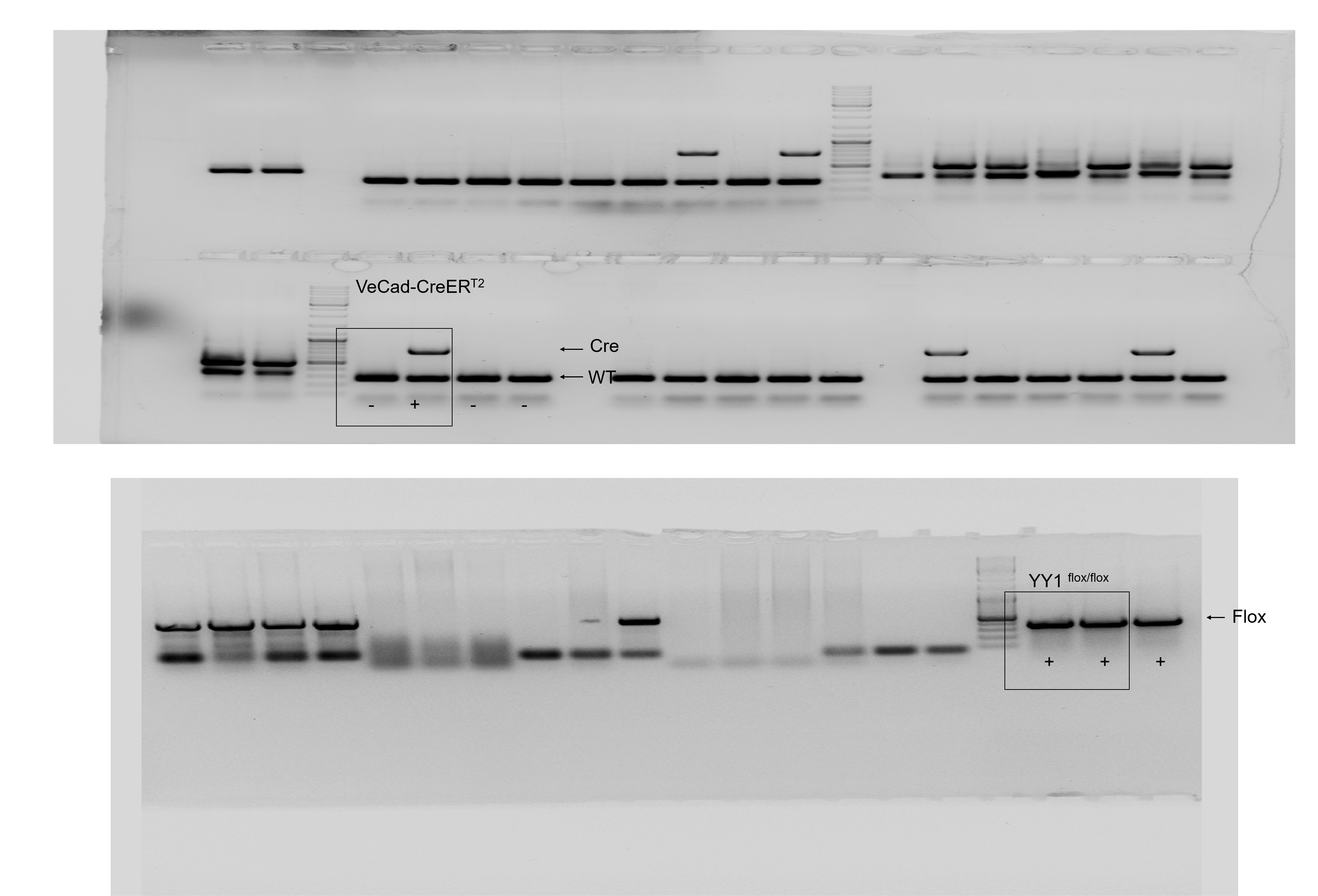


Figure S1: Full length Gels of VeCad-CreER^T2^ and YY1 ^flox/flox^ in mouse tail (shown as cropped images in Figure 2B).


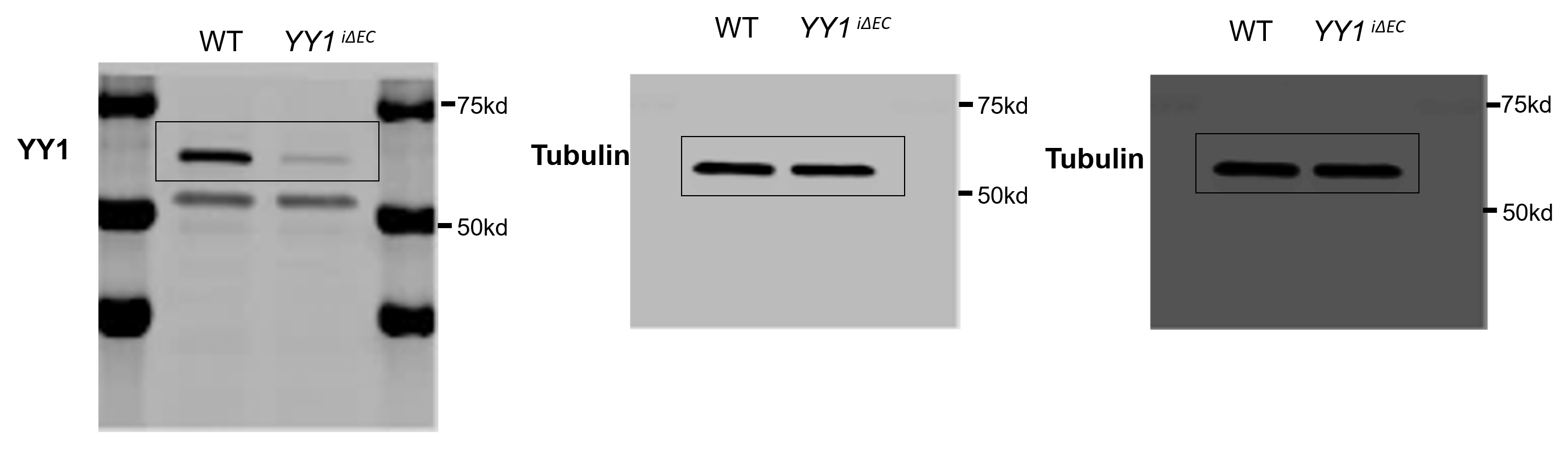


Figure S2: Full length blots of YY1 and tubulin in mouse lung endothelial cells isolated from WT and *YY1^iΔEC^* mice (shown as cropped images in Figure 2C). The nitrocellulose membrane after gel transferring was cut into a small piece. The same membrane was used for YY1 and tubulin immunoblotting. First, the membrane was incubated with YY1 antibody and then scanned by Licor Image System (left panel). The antibody was been striped by NaOH buffer and incubated with tubulin antibody. Due to very strong signals for the tubulin antibody immunoactivity, the lower exposure and small scan size was selected in Licor Image System (middle panel). While the protein markers did not react with tubulin antibody-associated IgG, but 75kd protein marker could still be identified by increasing the intensity of the image (right panel).


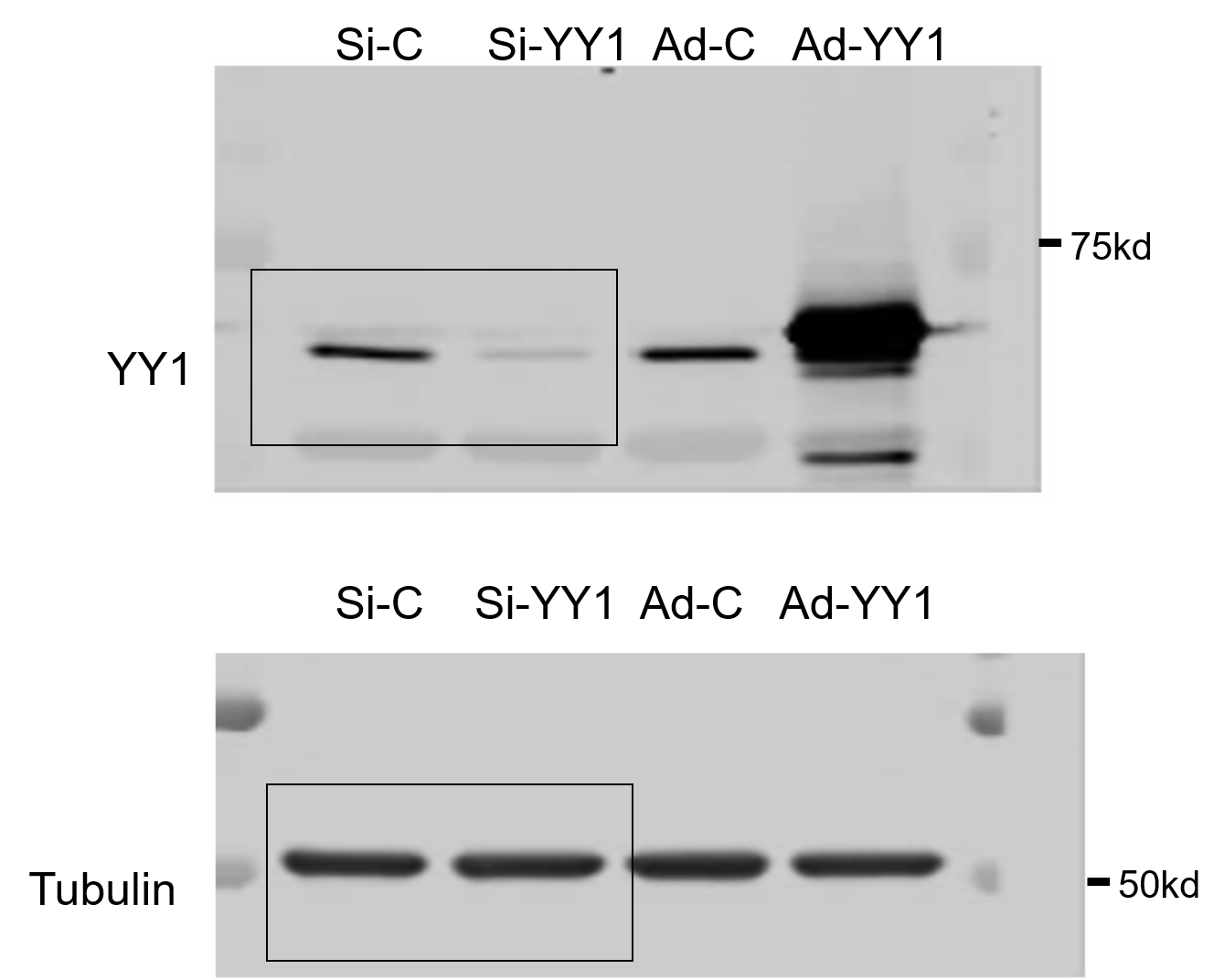


Figure S3: Full length blots of YY1 and Tubulin in HUVECs treated with YY1 siRNA (Si-YY1) and control siRNA (Si-C) for 24 hours. (shown as cropped images in Figure 6B).
